# Association Between BMI and Recurrence of Primary Spontaneous Pneumothorax

**DOI:** 10.1007/s00268-016-3848-8

**Published:** 2016-12-01

**Authors:** Juntao Tan, Yang Yang, Jianhong Zhong, Chuantian Zuo, Huamin Tang, Huimin Zhao, Guang Zeng, Jianfeng Zhang, Jianji Guo, Nuo Yang

**Affiliations:** 1 Department of Emergency, The First Affiliated Hospital of Guangxi Medical University, Shuang Yong Rd. #6, Nanning, 530021 People’s Republic of China; 2 Department of Hematology, The First Affiliated Hospital of Guangxi Medical University, Nanning, People’s Republic of China; 3 Department of Surgery Oncology, Affiliated Tumor Hospital of Guangxi Medical University, Nanning, People’s Republic of China; 4 Department of Cardiothoracic Surgery, The First Affiliated Hospital of Guangxi Medical University, Shuang Yong Rd. #6, Nanning, 530021 People’s Republic of China

## Abstract

**Background:**

Whether body mass index (BMI) is a significant risk factor for recurrence of primary spontaneous pneumothorax (PSP) remains controversial. The purpose of this study was to examine whether BMI and other factors are linked to risk of PSP recurrence.

**Methods:**

A consecutive cohort of 273 patients was retrospectively evaluated. Patients were divided into those who experienced recurrence (*n* = 81) and those who did not (*n* = 192), as well as into those who had low BMI (*n* = 75) and those who had normal or elevated BMI (*n* = 198). The two pairs of groups were compared in terms of baseline data, and Cox proportional hazards modeling was used to identify predictors of PSP recurrence.

**Results:**

Rates of recurrence among all 273 patients were 20.9% at 1 year, 23.8% at 2 years, and 28.7% at 5 years. Univariate analysis identified the following significant predictors of PSP recurrence: height, weight, BMI, size of pneumothorax, and treatment modality. Multivariate analyses identified several risk factors for PSP recurrence: low BMI, pneumothorax size ≥50%, and non-surgical treatment. Kaplan–Meier survival analysis indicated that patients with low BMI showed significantly lower recurrence-free survival than patients with normal or elevated BMI (*P* < 0.001).

**Conclusions:**

Low BMI, pneumothorax size ≥50%, and non-surgical treatment were risk factors for PSP recurrence in our cohort. Low BMI may be a clinically useful predictor of PSP recurrence.

## Introduction

Pneumothorax is defined as the presence of air or gases between the parietal and visceral pleural space. Pneumothorax can be clinically classified as either traumatic or spontaneous, and the spontaneous type can be further categorized as primary or secondary based on etiology [1]. Primary spontaneous pneumothorax (PSP) usually occurs in young, tall, and thin males without previous lung disease [2]. PSP is a relatively common disease with an annual incidence of 18–20/100,000 in males and 1.2–6.0/100,000 in females [3]. Treatment options for PSP involve surgical excision for bleb or bulla and conservative measures of observation, needle aspiration, and closed thoracostomy [4].

Even following optimal treatment to fully expand collapsed lungs, 20–60% of PSP patients suffer relapse, and this rate may be increasing [5]. Studies have suggested several risk factors for PSP recurrence, including age, gender, smoking, change in atmospheric pressure, emotional change, pneumothorax size, treatment modality, and body mass index (BMI) [1, 4]. BMI can serve as a measure of individual and community nutritional status [6]. Elevated BMI, known as overweight or obesity in extreme cases, is a strong risk factor for metabolic diseases, including coronary heart disease, hypertension, and diabetes mellitus [7, 8]. Low BMI, known as underweight, indicates malnutrition and nutrient deficiency, which may be associated with low numbers of immune cells and increased vulnerability to infectious diseases [8–10].

The possible association between BMI and risk of PSP recurrence remains controversial, with some studies suggesting a significant relationship [11, 12] but other studies failing to confirm this finding [5, 13]. In the present study, we retrospectively assessed the effect of low BMI on risk of PSP recurrence in a cohort of Chinese patients admitted to the thoracic surgery or emergency departments of a single large hospital. We hypothesized that underweight patients would be more likely to experience PSP recurrence than those with normal or elevated BMI.

## Materials and methods

The study protocol was approved by the Institutional Review Board of the First Affiliated Hospital of Guangxi Medical University (Nanning, China), and it was conducted in accordance with the Declaration of Helsinki. Medical records were retrospectively reviewed for patients with PSP admitted to the thoracic surgery or emergency departments of the First Affiliated Hospital of Guangxi Medical University between October 2010 and September 2014. Patients were enrolled in the study if (1) they fulfilled the diagnostic criteria of PSP based on chest radiography or computed tomography (CT), (2) they had no history of PSP, and (3) they were between 12 and 30 years old when PSP occurred. Patients were excluded from the study if they had secondary, traumatic, or iatrogenic pneumothorax.

### Data collection and patient allocation into groups

Data were collected on age, sex, height, weight, BMI, location and size of pneumothorax, and treatments. Pneumothorax size was quantified in terms of percentages as described [14]: pneumothorax size ≥50% was classified as large or extensive, while <50% was considered small or moderate. Recurrence of PSP was defined as repeat pneumothorax in the ipsilateral or contralateral pleural space occurring later than one month after treatment to achieve full expansion in the initial pneumothorax [5]. Patients were assigned to groups depending on whether they experienced PSP recurrence or not.

Patients were also assigned to groups depending on whether their BMI was <18.5 or ≥18 kg/m^2^. This cutoff corresponds to the World Health Organization (WHO) threshold for classifying people as underweight or as normal/overweight/obese [15]. Since we wanted to focus on underweight as a potential risk factor, we did not perform separate subgroup analyses of patients classified by the WHO as normal (BMI ≥18 and <25 kg/m^2^), overweight (BMI ≥25 and <30 kg/m^2^), or obese (BMI ≥30 kg/m^2^).

### PSP treatments

Treatments given to the patients in our cohort for the first PSP episode included oxygen therapy, needle aspiration, chest tube drainage, and video-assisted thoracoscopic surgery (VAST). The clinicians and techniques used to administer these treatments remained the same throughout the study period. Asymptomatic patients with small pneumothorax were treated by oxygen inhalation therapy; symptomatic patients with small pneumothorax were treated by chest tube drainage, as were symptomatic or asymptomatic patients with any other size of pneumothorax. VAST was administered to patients who experienced air leakage lasting 4–5 days, tension pneumothorax, hemopneumothorax, or occupational demand. Surgery was also used to treat patients with bullae visible on chest X-rays or CT scans, depending on the wishes of patients or their relatives.

### Follow-up

All patients were followed up after discharge until recurrence or October 2015. In principle, patients were followed up every one month during the first two years after discharge, and every 3–6 months thereafter. Long-term data on PSP recurrence were obtained through telephone interviews.

### Statistical analysis

Continuous data were expressed as mean ± standard deviation (SD), and intergroup differences were assessed for significance using the *t* test or Mann–Whitney *U* test. Categorical data were expressed as number (%), and intergroup differences were assessed using the Chi-squared or Fisher’s exact tests (2-tailed) as appropriate. Variables with a *P* value less than 0.05 in univariate analysis were entered in multivariate analysis. At the same time, height and weight that are closely related to BMI cannot be entered in multivariate Cox proportional hazards. BMI Kaplan–Meier survival curves were estimated and then compared using the log-rank test. The threshold of significance in all analyses was *P* < 0.05. All analyses were performed using SPSS 19.0 (IBM, USA).

## Results

### Patient characteristics

During the study period, 305 patients who experienced first PSP were admitted to our hospital and treated by conservative or surgical techniques. Excluded from the present series were 16 patients (5.2%) who had history of PSP, 7 patients (2.3%) with secondary pneumothorax, or 9 patients (3.0%) older than 30 years with PSP. Therefore, 273 patients (89.5%) were included in the analysis. Most patients (81.0%) were male, with a mean age of 19.8 years (SD 3.5). Average height was 173.6 ± 4.7 cm, and average weight was 59.2 ± 7.3 kg. The distribution of BMI was as follows: underweight, 27.5%; normal, 70.3%; overweight, 2.2%; and obese, 0%. Only 7.0% of patients had a smoking history. Location of pneumothorax was right in 53.1% of patients, left in 46.2%, and bilateral in 0.7%. Two patients who experienced bilateral pneumothorax underwent VAST, one of them had a relapse, and the other did not (Table 1).

**Table 1:** Demographic and clinical characteristics of patients with PSP, stratified by recurrence

Parameter	Total (*n* = 273) *N* (%)	Recurrence (*n* = 81)	Non-recurrence (*n* = 192)	*P* Re versus non-re
Age (years)	19.8 ± 3.5	19.4 ± 4.3	20.1 ± 4.6	0.242
Male, *n* (%)	221 (81.0%)	63 (28.5%)	158 (71.5%)	0.386
Height (cm)	173.6 ± 4.7	174.3 ± 4.1	173.0 ± 4.5	0.026
Weight (kg)				
BMI (kg/m^2^), *n* (%)	59.2 ± 7.3	57.1 ± 5.0	62.2 ± 6.9	<0.001
Underweight	75 (27.5%)	46 (61.3%)	29 (38.7%)	
Normal	192 (70.3%)	34 (17.7%)	158 (82.3%)	
Overweight	6 (2.2%)	1 (16.7%)	5 (83.3%)	
Obese	0 (0%)	0 (0%)	0 (0%)	
Smoking history, *n* (%)				0.850
Yes	19 (7.0%)	6 (31.6%)	13 (68.4%)	
No	254 (93.0%)	75 (29.5%)	179 (70.5%)	
Location of pneumothorax, *n* (%)				0.490
Right	145 (53.1%)	39 (26.9%)	106 (73.1%)	
Left	126 (46.2%)	41 (32.5%)	85 (67.5%)	
Bilateral	2 (0.7%)	1 (50%)	1 (50%)	
Size of pneumothorax, *n* (%)				0.002
<50	171 (62.6%)	27 (33.3%)	137 (71.4%)	
≥50	102 (37.4%)	54 (52.9%)	48 (47.1%)	
Treatment, *n* (%)				<0.001
Non-surgery	97 (35.5%)	57 (58.8%)	40 (41.2%)	
Surgery	176 (64.5%)	24 (13.6%)	152 (86.4%)	
Overall recurrence rate (year)				
1	57 (20.9%)	–	–	–
2	65 (23.8%)	–	–	–
5	81 (28.7%)	–	–	–

Values shown are mean ± SD or *n* (%)

*PSP* primary spontaneous pneumothorax, *Re* recurrence, *non-re* non-recurrence, *BMI* body mass index

Pneumothorax size was <50% in 62.6% of patients and ≥50% in 37.4%. Treatment was surgical in the case of 176 patients (64.5%) and non-surgical in the remaining 97 (35.5%). Among all 273 patients, 81 (29.7%) experienced recurrence and 192 did not. Rates of PSP recurrence were 20.9% at 1 year, 23.8% at 2 years, and 28.7% at 5 years (Table 1). Patients with or without recurrence did not differ significantly in age, gender, smoking history, or location of pneumothorax. The two groups did, however, differ significantly in height, weight, BMI, size of pneumothorax, and treatment modality (Table 1).

Patients were also classified into groups based on whether they were underweight (*n* = 75) or not (198). While the underweight group was slightly younger on average (19.3 ± 4.5 vs. 20.2 ± 4.8 years), the two groups did not differ significantly in gender, smoking history, location or size of pneumothorax, or treatment modality. The two groups did, however, differ significantly in height, weight, and PSP recurrence rates. Recurrence rates at 1, 2, and 5 years were 40.0, 48.0, and 58.7% in the underweight group, compared to 13.6, 15.2, and 18.7% in the normal/overweight group (Table 2).

**Table 2:** Demographic and clinical characteristics of patients with PSP, stratified by BMI

Parameter	BMI (kg/m^2^, *n* = 273)		
	<18.5 (*n* = 75) *N* (%)	≥18.5 (*n* = 198) *N* (%)	*P*
Age (years)	19.3 ± 4.5	20.2 ± 4.8	0.162
Male	57 (76.0%)	168 (84.9%)	0.380
Height (cm)	174.5 ± 4.9	172.8 ± 5.4	0.018
Weight (kg)	55.8 ± 5.5	62.8 ± 6.9	<0.001
Smoking history			0.343
Yes	7 (9.3%)	12 (6.1%)	
No	68 (90.7%)	186 (93.9%)	
Location of pneumothorax			0.704
Right	38 (50.7%)	107 (54.0%)	
Left	36 (48.0%)	90 (45.5%)	
Bilateral	1 (1.3%)	1 (0.5%)	
Size of pneumothorax (%)			0.159
<50	52 (69.3%)	119 (60.1%)	
≥50	23 (30.7%)	79 (39.9%)	
Treatment, *n* (%)			0.854
Non-surgery	26 (34.7%)	71 (35.9%)	
Surgery	49 (65.3%)	127 (64.1%)	
Overall recurrence rate (year)			
1	30 (40.0%)	27 (13.6%)	<0.001
2	35 (48.0%)	30 (15.2%)	<0.001
5	44 (58.7%)	37 (18.7%)	<0.001

Values shown are mean ± SD or *n* (%)

*PSP* primary spontaneous pneumothorax, *BMI* body mass index

### Risk factors for PSP recurrence

Univariate analysis identified the following significant prognostic factors for PSP recurrence (Table 1): height, weight, BMI, size of pneumothorax, and treatment modality. Multivariate analyses identified several risk factors for PSP recurrence (Table 3): BMI <18.5 kg/m^2^, pneumothorax size ≥50%, and non-surgical treatment.

**Table 3:** Multivariate analysis to identify predictors of PSP recurrence

Variable	Hazard ratio (95% CI)	*P*
BMI (<18.5 vs. ≥18.5 kg/m^2^)	3.668 (1.632–7.145)	<0.001
Pneumothorax size (≥50 vs. <50%)	1.571 (1.052–2.118)	0.017
Treatment modality (non-surgical vs. surgical)	1.317 (1.156–1.783)	0.038

*PSP* primary spontaneous pneumothorax, *BMI* body mass index

### Recurrence-free survival

**Fig. 1:**
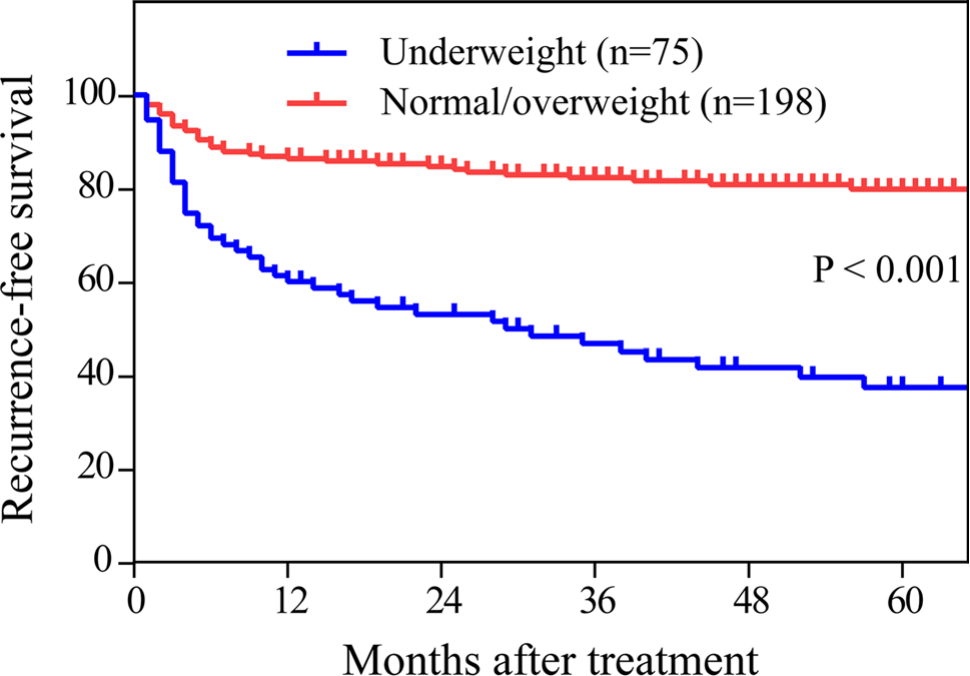
Kaplan–Meier analysis of recurrence-free survival in 273 patients with PSP, stratified by BMI (underweight vs. normal/overweight). The curves for the two groups differ significantly (*P* < 0.001)

Patients in our cohort were followed up for a median of 39 months (range 1–60). During follow-up, 44 patients in the underweight group (58.7%) relapsed, compared to 37 (18.5%) in the normal/overweight group. Rates of recurrence-free survival at 1, 2, and 5 years were 60, 52, and 41.3%, respectively, in the underweight group, which were significantly lower 1 than the rates of 86.4, 84.8, and 81.3%, respectively, in the normal/overweight group (*P* < 0.001, Fig. 1).

## Discussion

This retrospective cohort study associated low BMI (underweight) with risk of PSP recurrence and a lower rate of recurrence-free survival. These results suggest that BMI may be a helpful clinical indicator of PSP recurrence risk in patients experiencing their first pneumothorax.

Males predominated over females in our cohort by a ratio of 4:1, which is consistent, albeit slightly lower, than ratios of 9:1 reported in two Asian cohorts [13, 16]. Our univariate analysis identified height, weight, BMI, size of pneumothorax, and treatment modality as significant prognostic factors, which is consistent with other studies in European and Asian cohorts [11, 13, 17]. We did not detect a significant association between smoking and risk of PSP recurrence, even though it has been proposed to be a risk factor for first pneumothorax or PSP recurrence [18]. This may reflect the small proportion of smokers in our study (7.0%), which is smaller than previous work [19–21]. This suggests that anti-smoking public health campaigns in China have been effective and that people have greater health awareness.

Previous studies have reported conflicting results about whether BMI is associated with risk of PSP recurrence [11, 22, 23], and the discrepancy may reflect different inclusion criteria and small samples. Our results with a reasonably large cohort of 273 patients and adequate follow-up suggest that low BMI is indeed a risk factor for PSP recurrence, based on both uni- and multi-variate analyses, as well as Kaplan–Meier analysis of underweight and normal/overweight patients.

This association has several possible explanations. One is that most PSP patients with low BMI show unbalanced physical development. This significantly increases chest negative pressure, especially at the cupula of pleura, where it increases risk of bleb or bulla formation and pneumothorax [24]. A second possible explanation is that deficiencies in energy and nutrition associated with low BMI lead to deficiency in α_1_-antitrypsin. This leads, in turn, to major apical emphysema-like changes [25]. Deficiency in α_1_-antitrypsin was closely associated with bilateral bronchial abnormalities, which may be responsible for bilateral recurrences of PSP [5]. A third possible explanation is that abnormal bone mineral density in underweight PSP patients leads to rapid increases in body height and chest height [26], increasing chest negative pressure and therefore risk of PSP recurrence.

Risk of PSP recurrence may depend on several factors [27]. No matter underwent preventive surgery (VAST) or non-surgery treatment (aspiration, observation, or chest tube drainage), there was a recurrence risk of initial PSP. In this study, we have found that the rate of recurrence of PSP was significantly lower in preventive surgery than non-surgery treatment by univariate analyses (*P* < 0.001). The main reason may be that surgical excision for bleb or bulla indeed decreases the risk of recurrence of PSP. Consistent with this, our multivariate analysis identified pneumothorax size ≥50% and non-surgical treatment as independent predictors of PSP recurrence, in addition to low BMI. The hazard ratio associated with low BMI in our cohort was 3.668 (95% CI 1.632–7.145, *P* < 0.001), greater than the corresponding ratios associated with pneumothorax size ≥50% and non-surgical treatment. This suggests that BMI may be a clinically more important risk factor for PSP recurrence.

Kaplan–Meier survival curves were used to estimate PSP recurrence in patients stratified by BMI, and the result showed that low BMI showed significantly lower recurrence-free survival than patients with normal or elevated BMI (*P* < 0.001). This strengthens our finding that BMI may be a clinically useful indicator of recurrence risk. At the same time, our study has several limitations. First, it retrospectively analyzed patients from a single medical center, increasing risk of selection bias, which needed multicenter clinical trials to avoid such bias and verify the results. Second, most patients were high school and university students from the Guangxi Autonomous Region in western China, who are generally slightly shorter and thinner than Chinese from other parts of the country. Thus, our results may not be generalizable to other parts of China or to other countries. Third, we have failed to compare BMI at a later stage after initial treatment, the change of which may be of more sense to explain association between BMI and recurrence of PSP. Fourth, although we did examine several baseline characteristics as candidate risk factors for PSP recurrence, we may have failed to analyze other factors that are playing a role. Future studies should examine a more exhaustive set of possible risk factors.

Despite these limitations, our study provides evidence that low BMI is associated with higher risk of PSP recurrence and shorter recurrence-free survival. This work justifies larger clinical trials and mechanistic studies to elucidate how low BMI may influence PSP recurrence.
